# Bidirectional Transfer Study of Polystyrene Nanoparticles across the Placental Barrier in an *ex Vivo* Human Placental Perfusion Model

**DOI:** 10.1289/ehp.1409271

**Published:** 2015-05-08

**Authors:** Stefanie Grafmueller, Pius Manser, Liliane Diener, Pierre-André Diener, Xenia Maeder-Althaus, Lionel Maurizi, Wolfram Jochum, Harald F. Krug, Tina Buerki-Thurnherr, Ursula von Mandach, Peter Wick

**Affiliations:** 1 Laboratory for Particles-Biology Interactions, Empa, St. Gallen, Switzerland; 2 Perinatal Pharmacology, Department of Obstetrics, University Hospital Zurich, Zurich, Switzerland; 3 Graduate School for Cellular and Biomedical Sciences, University of Berne, Berne, Switzerland; 4 Institute of Pathology, Cantonal Hospital St. Gallen, St. Gallen, Switzerland; 5 Powder Technology Laboratory, Ecole Polytechnique Federale de Lausanne, Lausanne, Switzerland; 6 International Research Cooperations, Empa, St. Gallen, Switzerland

## Abstract

**Background:**

Nanoparticle exposure *in utero* might not be a major concern yet, but it could become more important with the increasing application of nanomaterials in consumer and medical products. Several epidemiologic and *in vitro* studies have shown that nanoparticles can have potential toxic effects. However, nanoparticles also offer the opportunity to develop new therapeutic strategies to treat specifically either the pregnant mother or the fetus. Previous studies mainly addressed whether nanoparticles are able to cross the placental barrier. However, the transport mechanisms underlying nanoparticle translocation across the placenta are still unknown.

**Objectives:**

In this study we examined which transport mechanisms underlie the placental transfer of nanoparticles.

**Methods:**

We used the *ex vivo* human placental perfusion model to analyze the bidirectional transfer of plain and carboxylate modified polystyrene particles in a size range between 50 and 300 nm.

**Results:**

We observed that the transport of polystyrene particles in the fetal to maternal direction was significantly higher than for the maternal to fetal direction. Regardless of their ability to cross the placental barrier and the direction of perfusion, all polystyrene particles accumulated in the syncytiotrophoblast of the placental tissue.

**Conclusions:**

Our results indicate that the syncytiotrophoblast is the key player in regulating nanoparticle transport across the human placenta. The main mechanism underlying this translocation is not based on passive diffusion, but is likely to involve an active, energy-dependent transport pathway. These findings will be important for reproductive toxicology as well as for pharmaceutical engineering of new drug carriers.

**Citation:**

Grafmueller S, Manser P, Diener L, Diener PA, Maeder-Althaus X, Maurizi L, Jochum W, Krug HF, Buerki-Thurnherr T, von Mandach U, Wick P. 2015. Bidirectional transfer study of polystyrene nanoparticles across the placental barrier in an *ex vivo* human placental perfusion model. Environ Health Perspect 123:1280–1286; http://dx.doi.org/10.1289/ehp.1409271

## Introduction

Currently the application of engineered nanoparticles (NP) in industrial and consumer products is increasing continuously. Epidemiological as well as *in vitro* studies have shown that engineered, naturally occurring, and combustion-derived NPs could have adverse health effects in humans (Bell et al. 2012; Pietroiusti 2012). However, to cause damage *in vivo*, NPs have to cross highly protective biological barriers. Besides the intestine and skin, the air–blood barrier of the lung is an important entry site for NPs. Multiple studies have shown that NPs are able to cross this protective barrier *in vitro* and *in vivo* (Geiser et al. 2005; Kreyling et al. 2009; Rothen-Rutishauser et al. 2007). Furthermore, NPs are applied in various medical products such as contrast agents for imaging or metal oxide particles for cancer therapy (Gupta and Gupta 2005; Rasmussen et al. 2010). Because these medical NPs are injected, they get direct access to the blood circulation. Therefore, it will be increasingly important to investigate NP transport across internal barriers such as the placental barrier between the mother and the unborn child.

The placenta is responsible for the supply of nutrients, the removal of waste products, and the protection of the fetus against harmful substances. It is organized in cotyledons, which represent the functional units of the placenta. Each cotyledon is formed by a fetal villous tree. Because of the extensive division of the villous trees, the total exchange surface area at term is about 13 m^2^ (Larsen et al. 1995; Syme et al. 2004). The maternal blood is released into the intervillous space and separated from the fetal circulation by the syncytiotrophoblast layer, some few cytotrophoblast cells, and the endothelial cell layer of the fetal capillaries, which are surrounded by stromal fibroblasts and fetal macrophages. The thickness of this barrier decreases during pregnancy to allow an increased maternal–fetal exchange at late gestational ages (Juch et al. 2013; Syme et al. 2004). The syncytiotrophoblast layer as a key barrier is formed by cytotrophoblast cells that fuse during development and form a true syncytium without lateral cell membranes (Enders and Blankenship 1999). The plasma membrane of the syncytiotrophoblast is highly polarized and consists of two membranes. The basal membrane is in contact with the villous stroma, which surrounds the fetal capillaries, and the brush border membrane, with its many microvilli, faces the maternal blood stream. The polarity of the syncytiotrophoblast is based on a different repertoire of transport proteins for each of these membranes. Furthermore, a huge variety of transporters act in both directions (importer and exporter) to ensure an optimal supply with nutrients and an efficient efflux of waste products or harmful drugs (Ganapathy et al. 2000). The placental transfer of such substances depends on four different mechanisms: passive diffusion, active transport, phagocytosis/pinocytosis, and biotransformation through metabolic enzymes (Syme et al. 2004).

Several animal studies showed that different NPs such as gold, silica, or titanium dioxide can cross the placental barrier, and some of them can even impair fetal development (Semmler-Behnke et al. 2008; Yamashita et al. 2011). However, the placenta is the most species-specific organ, and data obtained in rodent models cannot be simply extrapolated to the human system (Enders and Blankenship 1999; Takata and Hirano 1997). *Ex vivo* human placental perfusion provides an ethically accepted model, close to the *in vivo* situation, to investigate placental transport of xenobiotics as well as NPs (Grafmüller et al. 2013; Malek et al. 2009; Panigel et al. 1967; Schneider et al. 1972). Using this model, studies have found 25- and 50-nm silica particles to be transported across the human placental barrier, whereas pegylated 10- to 30-nm gold particles were retained in the maternal circulation and the placental tissue (Myllynen et al. 2008; Poulsen et al. 2015). Previous work performed by our group revealed a size-dependent translocation of polystyrene (PS) particles, with placental passage of PS particles up to 240 nm in diameter (Wick et al. 2010). Although the number of *in vivo* and *in vitro* studies about placental NP transport is increasing (Buerki-Thurnherr et al. 2012; Saunders 2009), the transport mechanisms for NPs across the placental barrier are largely unknown. Knowledge about the route of NP transport across the placental barrier and the dependency of this transport on the physicochemical properties of NPs is a prerequisite for future development of NPs as drug carriers to either specifically treat the mother without affecting the fetus or to treat placental dysfunctions. In addition, a better understanding of the translocation of NPs across an internal barrier such as the placenta would also contribute to a safer design of NPs in general. To assess the contribution of the physicochemical properties of NPs to placental NP transfer and the transport mechanisms underlying this process, we performed and analyzed bidirectional transfer studies of fluorescently labeled PS particles with different sizes and surface modifications in the *ex vivo* human placental perfusion model.

## Materials and Methods

*Particles.* We used plain (without functionalization) yellow-green–labeled PS beads of 50 and 240 nm (Spherotech). Yellow-green–labeled carboxylate-modified (COOH) 50- and 300-nm PS beads were purchased from Polysciences Inc.

*Particle characterization.* We determined the zeta potential in 10 mM sodium chloride and perfusion medium at pH 6.8–7.2 using a Zetasizer NanoZS (Malvern Instruments).

Particle size distribution in double distilled (DD) water and perfusion medium (PM) was determined by nanoparticle tracking analysis (NTA; NanoSight LM 20 System, software version 2.3; NanoSight Ltd.) as described previously (Hole et al. 2013). The composition of the PM is described in Supplemental Material, “*Ex vivo* human placental perfusion model.” The DD water and PM were filtered through a 0.02-μm Anotop® 25 syringe filter (Whatman GmbH) prior to analysis. For each particle size, the results were normalized to the area under the NP concentration/size curve.

We determined the detection limit of the PS beads’ fluorescence by a serial dilution (0.02–10 μg/mL) of each PS particle in perfusion medium. The detection limit was defined as the minimum concentration of PS particles in perfusion medium, which showed a significant increase in fluorescence intensity compared with perfusion medium alone.

To assess the stability of fluorescence, we analyzed the loss of fluorescence intensity after incubation of the PS beads in perfusion medium at 37°C for 3, 6, 24, 48, and 72 hr using a microplate reader (Biotek Synergy HT) with excitation and emission wavelengths of 485 and 528 nm. The leakage of fluorescence was assessed by measuring the fluorescence before and after filtration through a 0.1-μm syringe filter at the end of the indicated incubation periods.

*Cell culture.* BeWo cells (b30 clone), a cell line derived from human choriocarcinoma, were obtained from U. Graf-Hausner (Zurich University of Applied Sciences, Waedenswil, Switzerland) with permission from A.L. Schwartz (Washington University School of Medicine, MO, USA). BeWo cells cultured in Ham’s F-12K medium (Gibco, Thermo Fisher Scientific Inc.) supplemented with 1% penicillin-streptomycin and 10% fetal calf serum (FCS) at 37°C and 5% carbon dioxide (CO_2_).

*MTS viability assay.* The *in vitro* cytotoxicity of the different PS beads was tested using the MTS viability assay. Twenty-four hours before treatment, BeWo cells were seeded in a 96-well plate (8,000 cells/well) and different concentrations of PS beads were applied. Cells without treatment were used as the negative control, and 1, 10, 100, and 1,000 μM cadmium sulfate was used as the positive control. After 3 or 24 hr of incubation at 37°C and 5% CO_2_, an MTS assay (CellTiter96® AQ_ueous_ One Solution Cell Proliferation Assay; Promega) was performed according to the manufacturer’s instructions. Results are presented as the mean percentage of the untreated control from three independent experiments.

*Viability and functionality, antipyrine transfer, and histopathological evaluation of placental tissue.* Glucose and lactate concentrations in the fetal and maternal circuit were determined as indicators of tissue viability. Production of the placental hormones human chorionic gonadotropin and leptin were estimated to assess tissue functionality. Tissue samples of nonperfused and perfused placentas were fixed and examined by light microscopy. Detailed procedures are provided in Supplemental Material, “Histopathological evaluation.”

Ex vivo *human placental perfusion model.* The placentas were obtained from uncomplicated term pregnancies after cesarean section at the Department of Obstetrics, University Hospital Zurich and the Kantonsspital and the Klinik Stephanshorn in St. Gallen. Written informed consent was obtained prior to delivery. The project was approved by the local ethics committee and performed in accordance with the principles of the Declaration of Helsinki. The placenta perfusion was performed as described previously (Grafmüller et al. 2013; Wick et al. 2010). For a brief description see Supplemental Material, *“Ex vivo* human placenta perfusion model.”

*Fluorescence microscopy.* Unstained paraffin sections of nonperfused (negative control) and perfused placenta were deparaffinized with xylene followed by 100% ethanol. The slides were then air-dried and covered with VECTASHIELD® Mounting Medium containing DAPI (Vector Laboratories) on a glass slide; the coverslips were sealed with nail polish. The slides were analyzed with a Leica DM6000B fluorescence microscope system (Leica Microsystems) equipped with a triple band pass filter set (DAPI/Spectrum Green/Spectrum Orange).

*Transmission electron microscopy (TEM).* Particle suspensions, as supplied by the manufacturer, were applied onto a carbon-coated copper grid and processed for TEM analysis (Zeiss 900 TEM; Carl Zeiss MicroImaging). Samples from fetal or maternal circulation after 1.5–6 hr of perfusion were centrifuged twice for 30 min at 25,000 × *g* at 4°C. The pellets were resuspended in DD water and processed for TEM analysis as described for the particle suspensions.

*Statistical analysis.* Data are presented as mean ± SD of at least three independent experiments. Unpaired Student’s *t*-test was performed using GraphPad Prism software, version 6 (GraphPad Software). Differences were considered statistically significant at *p* < 0.05.

## Results

**Figure 1:**
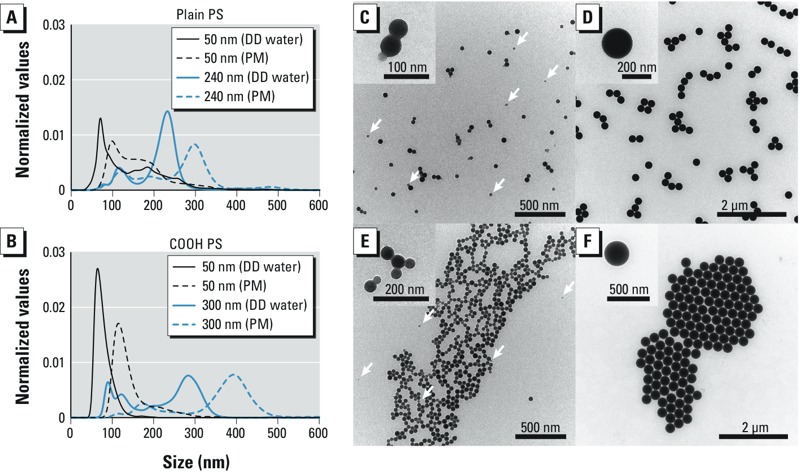
Particle size distribution (*A*,*B*) and TEM analysis (*C*–*F*) of PS beads. Size distribution of plain (*A*) and COOH (*B*) PS beads was measured in DD water and PM by nanoparticle tracking analysis. (*C*–*F*) TEM micrographs of plain 50-nm (*C*), plain 240-nm (*D*), 50-nm COOH (*E*), and 300-nm COOH (*F*) PS beads in DD water. Insets show a higher magnification of the PS beads. Arrows indicate an additional fraction of smaller PS beads. Abbreviations: COOH, carboxylate-modified; DD, double distilled; PM, perfusion medium; PS, polystyrene; TEM, transmission electron microscopy.

*Particle characterization and cytotoxicity evaluation.* The fluorescently labeled PS beads were extensively characterized, and the results are summarized in Table 1 and Figure 1. All PS particles suspended in 10 mM sodium chloride solution were negatively charged, but the zeta potential of the COOH PS beads was significantly lower than that of plain PS beads in the same size range. Analysis of the size distribution by nanoparticle tracking analysis confirmed that the plain 50-nm and COOH 50-nm PS beads were relatively monodisperse (Figure 1A,B). However, the plain 240-nm and COOH 300-nm PS beads contained an additional fraction of smaller beads around 100 nm, which were not observed to that extent in the corresponding TEM micrographs (Figure 1D,F). Furthermore, TEM images demonstrated that the plain and COOH 50-nm PS beads contained some smaller particles of around 20 nm in diameter (Figure 1C,E). As expected, the hydrodynamic diameter was higher for all PS beads in perfusion medium compared with those in water (Table 1).

**Table 1:** Summary of PS bead characteristics.

NP characteristic	Plain		COOH	
	50 nm	240 nm	50 nm	300 nm
Diameter (nm)^*a*^	49	240	42.5	302.7
Diameter in TEM (nm)^*b*^	43.7 ± 8	220.5 ± 5.1	44.1 ± 7.1	289.4 ± 10.2
Hydrodynamic diameter in DD water (nm)^*c*^	88 ± 79.5	230 ± 65.3	68 ± 19.2	283 ± 85.2
Hydrodynamic diameter in PM (nm)^*c*^	104 ± 74.7	273 ± 95.4	114 ± 49.1	359 ± 101.2
Initial no. of particles/mL in PM^*d*^	5.45 × 10^11^	4.24 × 10^9^	5.30 × 10^11^	1.88 × 10^9^
Particle surface (nm^2^)/mL in PM^*d*^	3.27 × 10^15^	6.48 × 10^14^	3.24 × 10^15^	4.94 × 10^14^
Detection limit in PM (μg/mL)	< 1.25	< 0.63	< 0.078	< 0.078
Zeta potential in 10 mM NaCl (mV)^*b*^	–19.8 ± 4.0	–20.5 ± 2.7	–34.7 ± 7.1*	–55.6 ± 6.1*
Zeta potential in PM (mV)^*b*^	–11.3 ± 6.5	–13.7 ± 5.8	–11.9 ± 11.2	–13.9 ± 7.4
Abbreviations: DD, double distilled; PM, perfusion medium; TEM, transmission electron microscopy. ^***a***^According to the manufacturer’s information. ^***b***^Experimentally determined (mean ± SD). ^***c***^Experimentally determined (mode ± SD). ^***d***^Calculated values. *p < 0.05 compared with plain beads of the same size.				

Because a few studies have shown a leakage of the fluorescence dye from NPs (Pietzonka et al. 2002; Tenuta et al. 2011), we tested their stability in perfusion medium over a time period of 72 hr at 37°C. After 3 hr, fluorescence intensity decreased to 75 ± 4% of the initial signal for the plain 50-nm, 76 ± 2% for the plain 240-nm, 83 ± 4% for the COOH 50-nm, and 84 ± 5% for the COOH 300-nm PS beads, but then remained stable up to 72 hr (see Supplemental Material, Figure S1A). This slight decrease in fluorescence intensity was not due to a loss of fluorescence dye from the particles, as mean leakage of the dye was < 0.52% for the plain 240-nm and 2.3% for the COOH 300-nm PS beads (see Supplemental Material, Figure S1B). Filtration of the smaller beads through a 20-nm syringe filter was attempted, but it failed due to the high viscosity of the perfusion medium and the obstruction of the filter. Therefore, leakage of the dye could not be assessed in these samples.

To confirm the absence of cytotoxic effects of the PS beads, an MTS viability assay was performed on BeWo cells. These cells were used as a model of the syncytiotrophoblast, which contact the NPs in the *ex vivo* perfusion system first. None of the PS particles significantly decreased cell viability, even at higher concentrations and longer exposure time than those used in the *ex vivo* perfusion experiments (see Supplemental Material, Figure S2).

**Figure 2:**
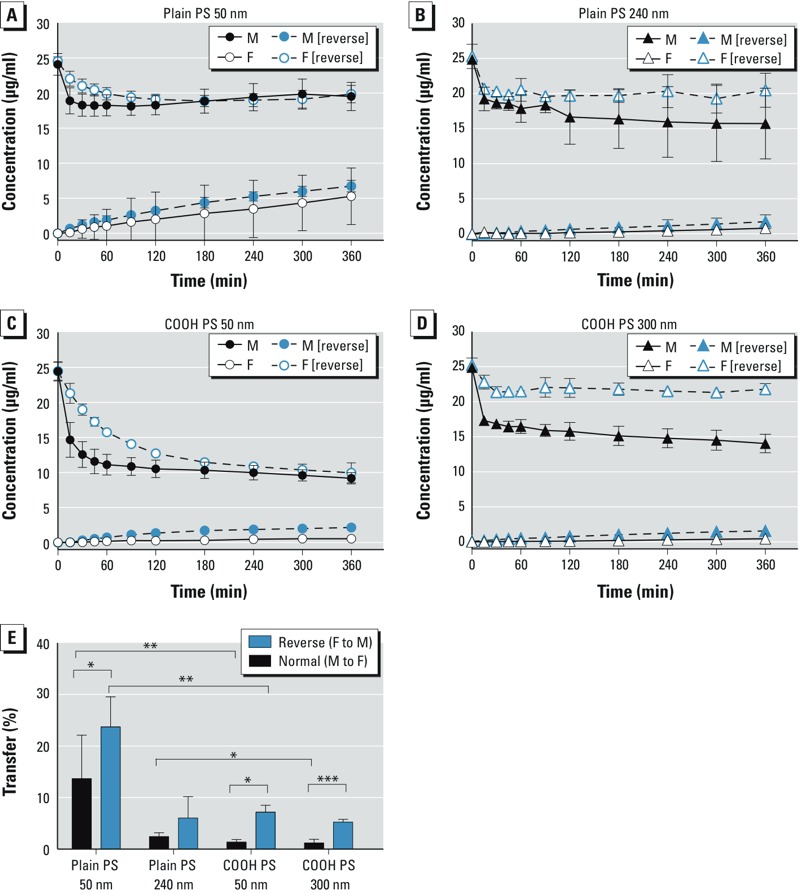
Perfusion profiles and transfer rates of PS beads during *ex vivo* human placental perfusion. Transplacental transport of plain (*A,B*) and COOH (*C,D*) PS beads over 6 hr either from maternal to fetal (M to F; normal) or from F to M (reverse) circulation. Beads (25 μg/mL) were added to the perfusions, and the concentration of particles was determined by fluorescence measurement at several time points. Data represent the particle concentration (mean ± SD) of at least three independent experiments. (*E*) Transfer of PS beads calculated after 6 hr of perfusion. Data are expressed as the percentage of the initial concentration of PS beads added (mean ± SD) of at least three independent experiments. Abbreviations: COOH, carboxylate-modified; PS, polystyrene. *p < 0.05. **p < 0.01. ***p < 0.001.

*Placental transfer.* In a previous study, we observed a size-dependent transfer of PS beads after 3 hr of *ex vivo* human placental perfusion in the maternal to fetal direction, with the highest transfer rate for plain 50-nm PS beads (Wick et al. 2010). In the present study we investigated the bidirectional placental transfer of plain 50- and 240-nm and COOH 50- and 300-nm PS particles by adding 25 μg/mL PS beads to the maternal (M) or to the fetal (F) circulation. After 6 hr of perfusion, the concentration of all PS beads was increased in reverse direction (F→M) compared with normal perfusions (M→F) (Figure 2A–D). We observed a significant difference in placental transfer between normal and reverse perfusions for the plain 50-nm (M→F, 13.7 ± 8.4% vs. F→M, 23.7 ± 5.8%), the COOH 50-nm (M→F, 1.4 ± 0.5% vs. F→M, 7.2 ± 1.3%), and COOH 300-nm PS beads (M→F, 1.2 ± 0.7% vs. F→M, 5.3 ± 0.5%) (Figure 2E). Plain 240-nm PS beads also showed a tendency for a higher transfer in the reverse direction (M→F, 2.4 ± 0.7% vs. F→M, 6.1 ± 4.1%), indicating a generally increased placental permeability in the F→M direction. In addition, we showed an increased translocation of 50-nm plain PS beads compared with COOH 50-nm PS particles in both directions, indicating that the surface charge or modification of NPs could also influence placental NP transfer (Figure 2A,C,E). For the particles in the size range between 240 and 300 nm, a significant difference between plain and COOH beads was also observed, but only in perfusions from the M→F direction (Figure 2B,D,E). To ensure that we did not measure placental transfer of detached fluorescence dye, we wanted to recover the PS beads from the fetal (in case of normal perfusions) or the maternal (reverse perfusions) perfusates after *ex vivo* perfusion. In TEM micrographs, both large (240 and 300 nm) and plain 50-nm PS beads were found in the maternal perfusate after reverse perfusions, whereas in the fetal perfusate after normal perfusions only the plain 50-nm PS beads were detected (see Supplemental Material, Figure S1C). Transfer of the plain 240-nm and both COOH beads from the M→F direction was too low (< 0.8 μg/mL) for detection by TEM, but still within the detection limit of the more sensitive fluorescence measurement. Moreover, during *ex vivo* placental perfusion, a large amount of other electron-dense substances such as proteins or sugars were released in both circulations, which made it especially difficult to find the small 50-nm PS beads via TEM analysis. Therefore, only the plain 50-nm PS beads were detectable in TEM micrographs due to their high transfer in both directions.

As a control for a passively transported substance across the placental barrier, we added radiolabeled ^14^C-antipyrine to each perfusion. After 4–6 hr, equal concentrations should be reached in both circulations (Challier et al. 1983), and F→M or M→F concentration ratios should be around 1. This criterion was fulfilled in all perfusions, demonstrating that the PS beads had no effect on barrier permeability itself (see Supplemental Material, Figure S3A,B). Of note, the development of the concentration equilibrium of antipyrine in reverse perfusions was decelerated compared with perfusions in the M→F direction (see Supplemental Material, Figure S3A,B). During the perfusion process, there was no influence of the PS beads on viability (glucose consumption and lactate production) and functionality (human chorionic gonadotropin and leptin secretion) of the placenta (see Supplemental Material, Figure S3C,D). Moreover, in histological tissue sections, we identified no visible structural changes to the placental tissue after perfusion with or without particles (data not shown).

**Figure 3:**
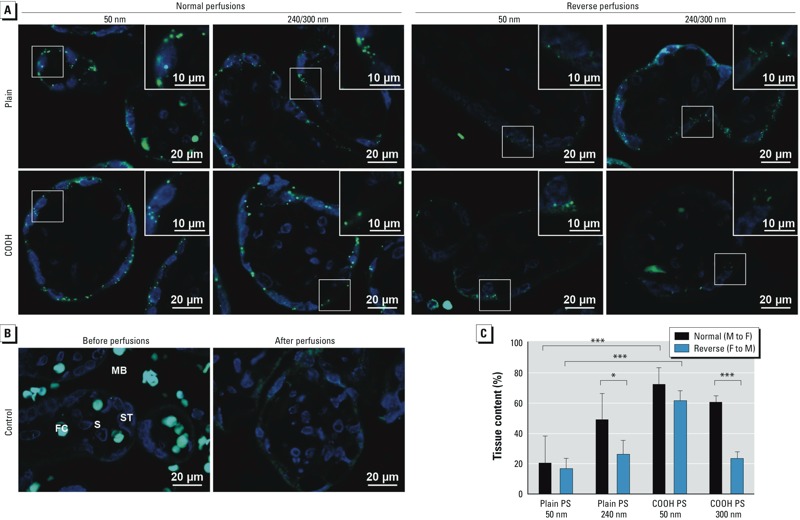
Localization (*A*,*B*) and quantification (*C*) of PS beads in the placental tissue. (*A*) Fluorescence microscope images of placental tissue after 6 hr of perfusion in normal (M to F) and reverse (F to M) directions with plain 50‑nm, plain 240‑nm, COOH 50‑nm, and COOH 300‑nm PS beads (green). Nuclei were stained with DAPI (blue). (*B*) Fluorescence microscope images of placental tissue before and after control perfusions without particles. (*C*) Theoretical NP tissue content after 6 hr of perfusion. Values represent the percentage of initially added PS beads remaining after subtraction of the PS fractions detected in fetal and maternal circuits after 6 hr (mean ± SD of at least three independent experiments). Abbreviations: COOH, carboxylate-modified; FC, fetal capillary; MB, maternal blood space; NP, nanoparticles; PS, polystyrene; S, stroma; ST, syncytiotrophoblast; TEM, transmission electron microscopy. *p < 0.05. ***p < 0.001.

Despite little transfer of the plain 240-nm and COOH 50-nm and 300-nm PS beads in both directions, the maternal (normal perfusions) or fetal (reverse perfusions) concentration of these beads declined (Figure 2B,C,D). Fluorescence microscopic images showed that these particles accumulated in the placental tissue (Figure 3). PS beads were found mainly in the syncytiotrophoblast layer of the placental villi independent of particle size, functionalization, or mode of perfusion (Figure 3A,B). Unfortunately, a reliable quantification of the PS beads in the tissue based on the fluorescence images was not possible because resolution is not sufficient to visualize single particles and small agglomerates, which would lead to an underestimation of NP tissue content. Therefore, we calculated the theoretical concentration of PS beads in the tissue by subtracting the measured concentration in the fetal and maternal circuit from the initial concentration added (Figure 3C). After 6 hr of perfusion, the tissue content of the PS beads with a higher transfer rate (plain 50 nm, F→M and M→F; plain 240 nm, F→M; COOH 300 nm, F→M) was significantly lower compared with the PS beads with low placental transfer (COOH 50 nm, M→F and F→M; plain 240 nm, M→F; COOH 300 nm, M→F) (Figure 3C).

## Discussion

In this study we observed a bidirectional transfer of plain and COOH PS beads up to a size of 300 nm using the *ex vivo* human placental perfusion model. Placental transport was increased in reverse perfusions from the fetal to the maternal side, indicating that there are different transport mechanisms for PS particles on the fetal and maternal sides of the human placenta. Although transport in the reverse direction is physiologically not relevant regarding *in vivo* exposure to NPs, because it occurs only in the maternal circulation, reverse *ex vivo* placental perfusion is a common method to evaluate the mode of transport of many drugs across the human placenta (Nanovskaya et al. 2012; Nekhayeva et al. 2005; Sudhakaran et al. 2005). For example, a study about bidirectional placental transfer of antibiotics revealed that telavancin had a higher placental transfer in the reverse direction, suggesting that a translocation occurred by means other than passive diffusion and indicating that specific transporters may be involved (Nanovskaya et al. 2012). However, it is rather unlikely that NPs, in general, are transported via such transporters across the placenta (Menezes et al. 2011). Nevertheless, our results demonstrate that passive diffusion is not the key mechanism underlying placental translocation of PS particles because concentration equilibrium was not achieved after 4–6 hr compared with the passively transported antipyrine. According to Fick’s law, diffusion of a substance depends mainly on the permeability of the membrane for the specific substance, the concentration gradient across the membrane, and the membrane surface area. In the present study, placental transfer kinetics of antipyrine from the F→M direction was significantly delayed compared with the M→F direction; this was likely due to the lower exchange surface on the fetal side (i.e., the inner surface of the fetal capillaries compared with the very large brush border membrane of the syncytiotrophoblast on the maternal side) and the reduced fetal perfusion flow (3–4 mL/min compared with 12 mL/min for the maternal circuit) (Challier et al. 1983). Thus, NP transfer in the reverse direction should be reduced if it is based predominantly on a passive transport mechanism.

In contrast to such an expectation, we observed an augmented transfer in the F→M direction (compared with M→F) of PS particles independent of their physicochemical properties, suggesting an energy-dependent transport mechanism for PS particle translocation across the human placenta. Phagocytosis is one example of an energy-dependent mechanism proposed for NP uptake into cells, especially in phagocytes (Jud et al. 2013). Primary human macrophages have been shown to engulf COOH PS beads via this pathway, whereas THP-1 cells, a human monocytic leukemia cell line, use an endocytosis pathway (Lunov et al. 2011). During phagocytosis, vesicles with a diameter > 0.5 μm are formed, whereas the diameter of endocytotic vesicles is considerably smaller (Aderem and Underhill 1999). Caveolin-coated vesicles are defined as membrane invaginations with a diameter of 60–80 nm, and vesicles arising from clathrin-dependent endocytosis have a diameter of approximately 120–150 nm in human epithelial cells (McMahon and Boucrot 2011; Parton and Simons 2007). A study using specific transport pathway inhibitors revealed that A549 cells, a human alveolar epithelial cell line, take up gold NPs with a diameter of 15 nm mainly by endocytosis (Brandenberger et al. 2010). Moreover, endocytosis and transcytosis have also been proposed as the most common transport mechanisms for NPs at the blood–brain barrier (Kreuter 2014).

Most of the studies about NP uptake mechanism have been performed on nonpolarized cells or cell lines, which do not resemble a typical polarized barrier such as the placental syncytiotrophoblast. The different membrane properties and receptor repertoires on the apical and basal sides may allow different transport mechanisms depending on the site of exposure. So far, most groups have observed NP uptake in the syncytiotrophblast after *ex vivo* human placental perfusion even if NP translocation to the fetal circulation was absent or below the detection limit (Menjoge et al. 2011; Myllynen et al. 2008; Poulsen et al. 2015). We also observed that most of the PS particles accumulated predominantly in the syncytiotrophoblast layer, which indicates that the syncytium is a major determinant of NP transfer. Besides the syncytiotrophoblast, the endothelial cells of the fetal capillaries are also part of the placental barrier. These cells contribute to the barrier function and act primarily as a molecular sieve for larger hydrophilic molecules (Firth and Leach 1996). However, to evaluate the definite contribution of the fetal endothelium to placental NP transfer, *in vitro* co-culture models including trophoblasts, as well as endothelial cells, are necessary and currently under development (Levkovitz et al. 2013).

Nanoparticle uptake into cells also depends on the physicochemical properties of the materials (Lunov et al. 2011). We demonstrated that COOH PS beads were transferred across the placenta in significantly lower amounts than plain particles. The COOH PS beads had a lower zeta potential than the plain beads, indicating that the surface charge of NPs can have an impact on placental transfer. Indeed, such a surface charge–dependent placental translocation has been demonstrated in pregnant rats, where amine-modified PS beads showed a stronger translocation than COOH PS beads (Tian et al. 2009). Similar observations have been made with NPs at other biological barriers. The accumulation of negatively charged gold NPs in secondary organs after oral exposure in rats was higher than that of positively charged particles (Schleh et al. 2012). Furthermore, studies of NP uptake at the air–blood barrier revealed that the surface charge of NPs below a size threshold of 34 nm is the most critical factor for translocation (Choi et al. 2010). Differently charged NPs acquire a distinct protein corona after contact with serum or biological fluids (Hirsch et al. 2013; Lundqvist et al. 2008; Monopoli et al. 2011).

The protein corona can influence the biological fate of NPs through alteration of their hydrodynamic diameter or surface properties. In addition, serum proteins can also directly influence the uptake mechanism of NPs by binding to their specific receptors on the cell surface, thereby mediating endocytosis (Monopoli et al. 2012). Several groups observed that the albumin concentration in the perfusion medium determines the transplacental transfer of several drugs in the *ex vivo* human placental perfusion model (Mathiesen et al. 2009; Nanovskaya et al. 2008). The perfusion medium used in our study was supplemented with albumin only, not with complete serum. However, many other proteins are produced by placental cells during perfusion and are released into the circulation, where they can make contact with the PS beads. Interestingly, many hormones produced in the placenta are secreted asymmetrically into the maternal and fetal circulation (Linnemann et al. 2000; Malek et al. 2001). Therefore, adsorption of different proteins in fetal and maternal circulation may also provide an explanation for the differential transport in normal and reverse perfusions in our study. To corroborate this hypothesis, further studies are needed that examine both a broad variety of differently charged NPs and the composition of the NP protein corona.

Studies using the *ex vivo* human placental perfusion model are limited to a few hours of perfusion due to tissue degradation (Panigel et al. 1967; Schneider et al. 1972) and only reflect placental transport at late pregnancy. To assess long-term effects of NP exposure and transport during early stages of pregnancy, when the placental barrier is much thicker (Nikitina et al. 2015), *in vitro* studies are indispensable. In addition, *in vitro* studies would allow a higher throughput than *ex vivo* perfusions. Thus, involvement of specific transport pathways could be tested first *in vitro* and then be subsequently confirmed in the *ex vivo* placental perfusion model, which is more similar to the *in vivo* situation. Overall, development of more advanced and carefully validated *in vitro* models, which include flow cell systems and several placental cell types, are expected to lead to a better understanding of NP transport mechanisms across the placental barrier and their dependence on the physicochemical properties of NPs.

## Conclusions

To our knowledge, this is the first study to investigate the transport mechanism of NPs by examining the bidirectional transfer of PS particles in the *ex vivo* human placental perfusion model. We demonstrated an increased transfer of PS beads in reverse perfusions (F→M direction) and an accumulation of PS beads in the syncytiotrophoblast layer of the placental tissue. Based on our findings, we can exclude a transfer via passive diffusion. We propose an energy-dependent placental translocation pathway with the polarized syncytiotrophoblast as the main contributor to NP transfer in the placenta. For the development and the safe use of NPs in nanomedicine, transport mechanisms of NPs across the placental barrier need to be determined precisely in further studies.

## Supplemental Material

(6.9 MB) PDF
