# Supplementary material for: Bidirectional Transfer Study of Polystyrene Nanoparticles across the Placental Barrier in an ex Vivo Human Placental Perfusion Model
Source: Environ Health Perspect. 2015 May 8;123(12):1280–6. doi: 10.1289/ehp.1409271 (PMC4671239; doi:10.1289/ehp.1409271)
Supplement: (6.9 MB) PDF [file ehp.1409271.s001.acco.pdf]

Environ Health Perspect
doi: 10.1289/ehp.1409271

Note to Readers: *EHP* strives to ensure that all journal content is accessible to all readers. However, some figures and Supplemental Material published in *EHP* articles may not conform to 508 standards due to the complexity of the information being presented. If you need assistance accessing journal content, please contact ehp508@niehs.nih.gov. Our staff will work with you to assess and meet your accessibility needs within 3 working days.

Supplemental Material

Bidirectional Transfer Study of Polystyrene Nanoparticles across the Placental Barrier in an *ex Vivo* Human Placental Perfusion Model

Stefanie Grafmueller, Pius Manser, Liliane Diener, Pierre-André Diener, Xenia Maeder-Althaus, Lionel Maurizi, Wolfram Jochum, Harald F. Krug, Tina Buerki-Thurnherr, Ursula von Mandach, and Peter Wick

Table of Contents

Materials and Methods
Cell culture
MTS viability assay
Ex vivo human placental perfusion model
Antipyrine transfer
Viability and functionality of the placenta
Histopathological evaluation
References
Figure S1. Stability (A) and leakage (B) of the fluorescence dye was assessed after 3, 6, 24, 48 and 72 hrs of incubation at 37°C in perfusion medium. Shown are the mean percentages of initial (time point 0) fluorescence intensity ± SD (A) and the free fluorescence of the large PS particles

as percentage of total fluorescence intensity ± SD (B), n=3. (C) Representative TEM micrographs of plain 50 nm, 240 nm and carboxylate-modified 300 nm PS beads in the fetal or maternal circulation after 1.5 - 6 hrs of normal (M to F direction) or reverse (F to M direction) perfusion.

Figure S2. BeWo cell viability after 3 (A) and 24 hrs (B) of exposure to PS beads measured with a MTS assay. $CdSO_4$ served as positive control for cytotoxicity. Data represent the mean percentages of viable cells compared to the untreated control ± SD of at least 3 independent experiments.

Figure S3. (A + B) Fetal-to-maternal (F/M) or maternal-to-fetal transfer ratio (M/F) of radiolabeled ^{14}C-antipyrine over a time period of 6 hrs of normal (A) or reverse (B) placenta perfusion with or without PS beads. (C + D) Glucose consumption and lactate production (C) and human chorionic gonadotropin (hCG) and leptin hormone production rate (D) during *ex vivo* human placental perfusion with or without particles (control). All data is expressed as the mean ± SD of at least 3 independent experiments.

Materials and Methods

Cell culture

BeWo cells (b30 clone), a cell line derived from human choriocarcinoma, were obtained from Prof. Dr. Ursula Graf-Hausner (Zurich University of Applied Sciences, Waedenswil, Switzerland) with permission of Dr. Alan L. Schwartz (Washington University School of Medicine, MO, USA) and cultured in Ham´s F-12K medium (Gibco, Thermo Fisher Scientific Inc.) supplemented with 1 % penicillin-streptomycin and 10 % fetal calf serum (FCS) at 37°C and 5 % carbon dioxide (CO_2).

MTS viability assay

The *in vitro* cytotoxicity of the different PS beads was tested using the MTS viability assay. 24 hrs before treatment BeWo cells were seeded in a 96-well plate (8000 cells per well). Different concentrations of PS beads were applied. As negative control, cells without treatment were used and as positive control 1, 10, 100 and 1000 µM $CdSO_4$ was applied. After 3 or 24 hrs of incubation at 37 °C and 5 % CO_2, an MTS assay (CellTiter96® AQ_{ueous} One Solution Cell Proliferation Assay; Promega) was performed according to the manufacturer's instructions. Results were presented as mean percentage of the untreated control from three independent experiments.

Ex vivo human placental perfusion model

A fetal artery and vein of an intact cotyledon were cannulated. Afterwards the placenta was fixed in a tissue holder and placed into a perfusion chamber. To connect the maternal side three blunt cannulas were introduced in the intervillous space and a venous drain was introduced to return the fluid to the maternal circuit. Two peristaltic pumps maintained the fetal flow at 3 - 4 mL/min

and the maternal flow at 12 mL/min. A water bath kept the temperature of both circuits at 37 °C. For the fetal side one oxygenator with 95 % N_2 and 5 % CO_2 and for the maternal side one oxygenator with 95% synthetic air and 5 % CO_2 were applied. To flush out the blood and allow a recovery of the tissue from the ischemic period after the delivery, the experiment started with an open perfusion (both circuits) for 20 minutes using only perfusion medium. The perfusion medium contained M199 tissue culture medium (Sigma) diluted with Earl's buffer (dilution 1 : 2), 1 g/L glucose (Sigma), 10 g/L bovine serum albumin (AppliChem GmbH), 10 g/L dextran 40 (Sigma), 2500 IU/L sodium heparin (B.Braun Medical AG), 250 mg/L amoxicillin (GlaxoSmithKline AG) and 2.2 g/L sodium bicarbonate (Merck). After closing of the fetal and maternal circulation by leading the venous outflow back to the corresponding reservoir, the PS particles were added either to the maternal (M) or the fetal (F) reservoir at a final concentration of 25 μg/mL. The concentration of the PS beads in the fetal and maternal circuit was determined by fluorescence measurement in a microplate reader (Biotek Synergy HT) after centrifugation at 800 x g to remove residual erythrocytes. Particle concentrations were corrected for the PM volume in the tubes and volume loss due to sampling before placental transfer was calculated as percentage of transferred PS beads compared to the initially added particle amount. As control radiolabeled ^{14}C-antipyrine (50 nCi/mL, specific activity: 4.7 mCi/mmol; American Radiolabeled Chemicals Inc.) was also added to the maternal or fetal circuit. The criteria for a successful perfusion were visual control (intact membranes, no lesion, no disruption of the placenta), leak from fetal to maternal side < 4 mL/hr (for reverse perfusions < 1 mL/hr), an equilibrium of ^{14}C-antipyrine between maternal and fetal circuit after 4 - 6 hrs, a fetal perfusion pressure < 70 mmHg and fetal pH at a physiological range of 7.2 - 7.4.

Antipyrine transfer

Perfusate samples were mixed with scintillation cocktail (Irgasafe Plus Scintillation Cocktail, Zinsser Analytic) and measured in a liquid scintillation analyzer (Packard Tri-Carb 2200; GMI).

Viability and functionality of the placenta

Glucose and lactate concentration in the fetal and maternal circuit were determined with an automated blood gas system (ABL800 FLEX automated benchtop analyzer, Radiometer Medical ApS). The placental hormones human chorionic gonadotropin (hCG) and leptin in the fetal and maternal circuit before and after perfusion were determined by enzyme-linked immunosorbent assay (ELISA) as described previously (Malek et al. 1997; Malek et al. 2001). The hormone production was calculated by dividing the amount released in both circulations after 6 hrs of perfusion by the weight of perfused tissue.

Histopathological evaluation

Tissue samples of non-perfused (negative control) and perfused placentas were fixed in 4 % formaldehyde (Formafix AG). After standardized dehydratation of the tissue overnight in a Medite Tissue Processor TPC 15 the probes were embedded in paraffin blocks with the Medite Tissue Embedding System TES 99. Using a microtome, 4 µm thick sections were obtained, mounted and air dried on glass slides and stained with haematoxylin and eosin in a Tissue-Tek® Prisma™. The slides were examined at a Leica DMLB microscope.

References

Malek A, Sager R, Lang AB, Schneider H. 1997. Protein transport across the in vitro perfused human placenta. American journal of reproductive immunology 38:263-271.

Malek A, Willi A, Muller J, Sager R, Hanggi W, Bersinger N. 2001. Capacity for hormone production of cultured trophoblast cells obtained from placentae at term and in early pregnancy. Journal of assisted reproduction and genetics 18:299-304.

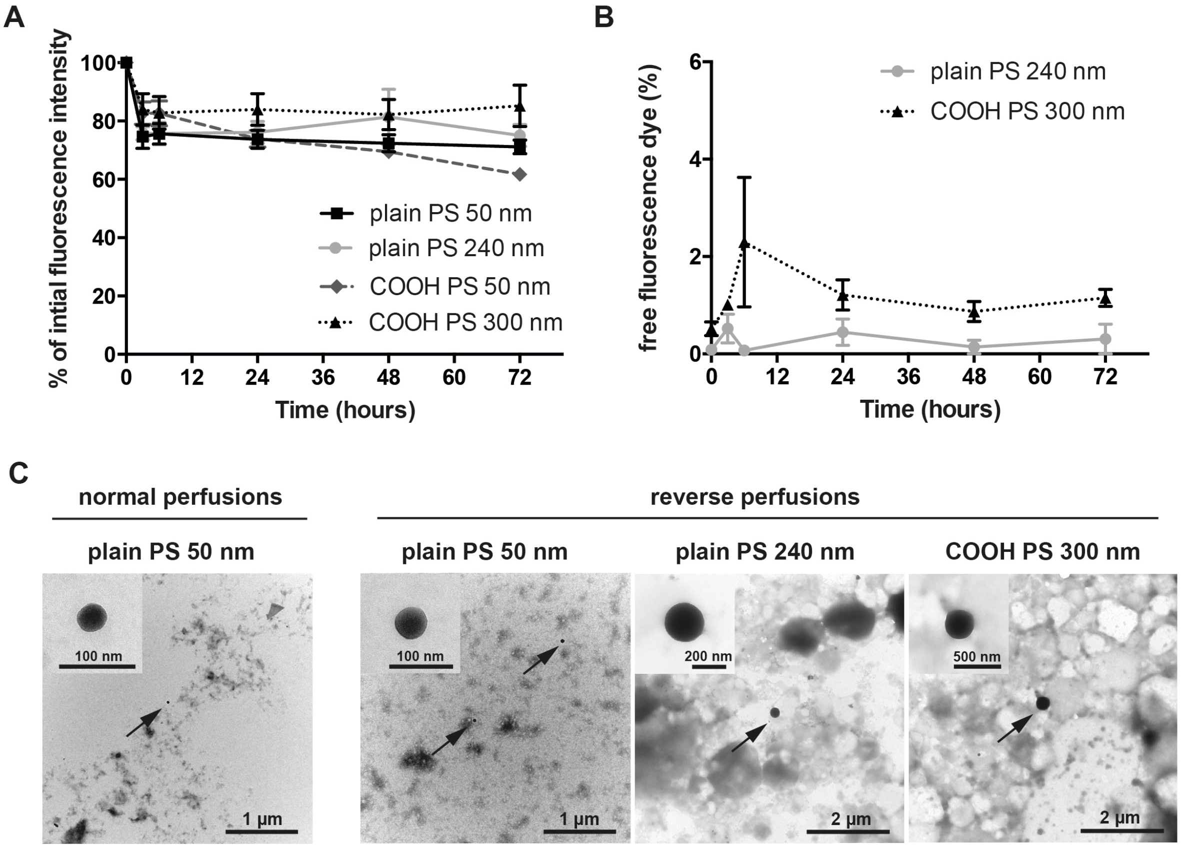

Figure S1. Stability (A) and leakage (B) of the fluorescence dye was assessed after 3, 6, 24, 48 and 72 hrs of incubation at 37°C in perfusion medium. Shown are the mean percentages of initial (time point 0) fluorescence intensity ± SD (A) and the free fluorescence of the large PS particles as percentage of total fluorescence intensity ± SD (B), n=3. (C) Representative TEM micrographs of plain 50 nm, 240 nm and carboxylate-modified 300 nm PS beads in the fetal or maternal circulation after 1.5 - 6 hrs of normal (M to F direction) or reverse (F to M direction) perfusion.

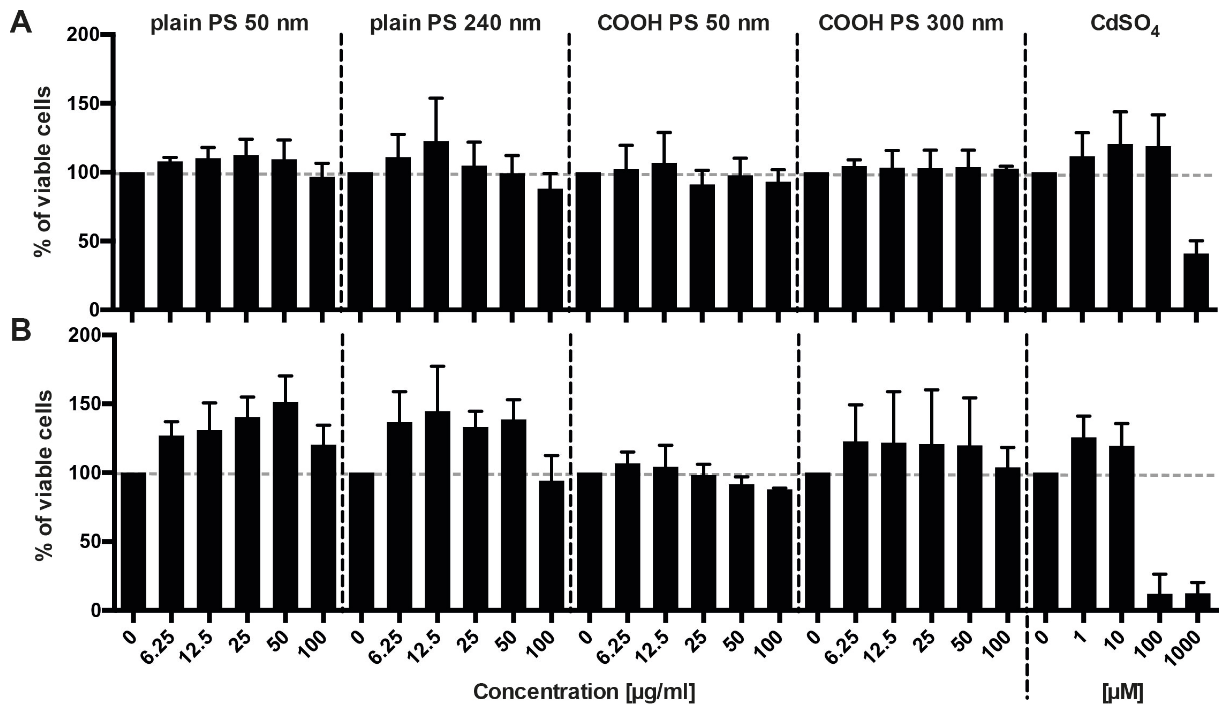

Figure S2. BeWo cell viability after 3 (A) and 24 hrs (B) of exposure to PS beads measured with a MTS assay. $CdSO_4$ served as positive control for cytotoxicity. Data represent the mean percentages of viable cells compared to the untreated control ± SD of at least 3 independent experiments.

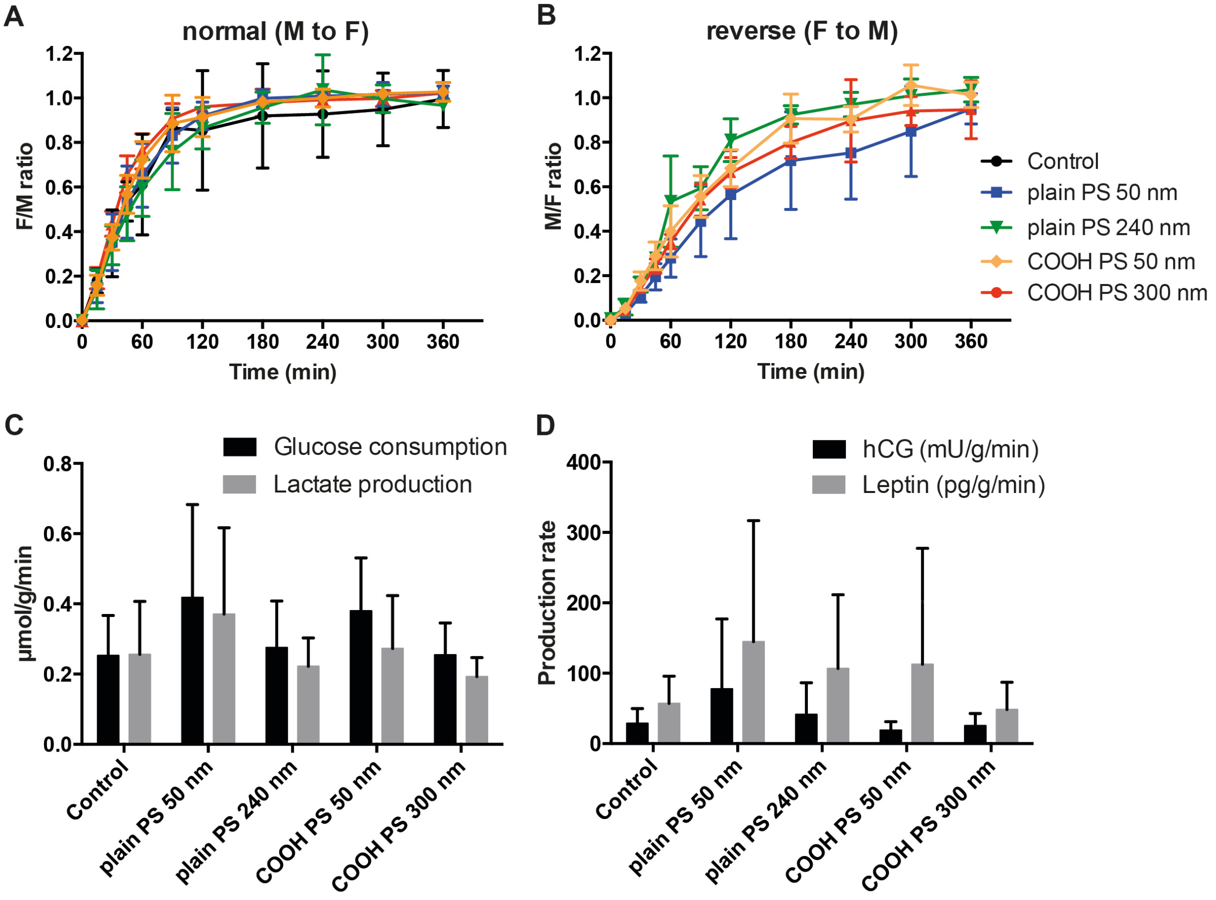

Figure S3. (A + B) Fetal-to-maternal (F/M) or maternal-to-fetal transfer ratio (M/F) of radio-labeled ^{14}C-antipyrine over a time period of 6 hrs of normal (A) or reverse (B) placenta perfusion with or without PS beads. (C + D) Glucose consumption and lactate production (C) and human chorionic gonadotropin (hCG) and leptin hormone production rate (D) during *ex vivo* human placental perfusion with or without particles (control). All data is expressed as the mean ± SD of at least 3 independent experiments.
